# Dosimetric quality and delivery efficiency of robotic radiosurgery for brain metastases: Comparison with C‐arm linear accelerator based plans

**DOI:** 10.1002/acm2.12746

**Published:** 2019-10-03

**Authors:** Shuming Zhang, Ruijie Yang, Xin Wang

**Affiliations:** ^1^ Department of Radiation Oncology Peking University Third Hospital Beijing China; ^2^ Department of Radiation Physics The University of Texas MD Anderson Cancer Center Houston TX USA

**Keywords:** CyberKnife, dosimetry, IMRT, multiple brain metastases, non‐coplanar, VMAT

## Abstract

The incidence of brain metastases is increasing and various treatment modalities exist for brain metastases. The aim of this study was to investigate the dosimetric quality and delivery efficiency of robotic radiosurgery (CyberKnife) for multiple brain metastases compared with C‐arm linear accelerator (linac) based plans. C‐arm linac based plans included intensity‐modulated radiation therapy (IMRT), volumetric modulated arc therapy (VMAT) and non‐coplanar VMAT with 1, 3 and 5 non‐coplanar arcs, respectively (NC1, NC3 and NC5). For 20 patients, six plans with a prescription dose of 30 Gy in three fractions were generated. The gradient index (GI), conformity index (CI), maximum dose (D_max_) of organs at risk (OARs), normal brain tissue volume (V_3 Gy_–V_24 Gy_), monitor units (MUs) and beam on time (BT) were evaluated. The GI of CyberKnife plans (3.60 ± 0.70) was lower than IMRT (6.21 ± 2.26, *P* < 0.05), VMAT (6.04 ± 1.93, *P* < 0.05), NC1 (5.16 ± 1.71, *P* < 0.05), NC3 (5.02 ± 1.59, *P* < 0.05) and NC5 (5.03 ± 1.72, *P* < 0.05). The CI of the VMAT plans (both coplanar and non‐coplanar) was larger than IMRT and CK plans. The D_max_ for most OARs of the CyberKnife plan was lower than the C‐arm linac based plans, although some differences were not statistically significant. The normal brain tissue volume of CyberKnife plan was lower than the C‐arm linac based plans, and the normal brain tissue volume of non‐coplanar VMAT plans was lower than IMRT and VMAT plans at high‐moderate dose level. However, the MUs and BT of CyberKnife plans was more than C‐arm linac based plans. CyberKnife plan was better than C‐arm linac based plans in protecting normal brain tissue and OARs for patients with multiple brain metastases. C‐arm linac based plan with non‐coplanar arc provided better protection of normal brain tissue than coplanar plan. However, the BT of CyberKnife plan was longer than C‐arm linac based plans.

## 1 INTRODUCTION

Due to improved outcome of systemic therapy against primary cancers and more sophisticated examination, up to 40% of patients with systemic cancer have brain metastases.^1^ At the same time, the overall incidence of brain metastases is also increasing.

Various treatment modalities exist for brain metastases, such as surgical resection, chemotherapy, molecular targeted therapy, whole‐brain radiotherapy (WBRT), stereotactic radiosurgery (SRS) and hypofractionated stereotactic radiotherapy (HFSRT). WBRT, a traditional technique for treating brain metastases, has been proved to increase cognitive decline without improving survival.^2^, ^3^ Sahgal et al^2^ found that the routine use of SRS + WBRT remained debatable when compared with SRS alone. Although WBRT showed greater distant brain control rates, it showed no effect on survival and increased adverse impact on patient cognition and quality of life.

However, SRS increases risk of neurological morbidity from radionecrosis.^4^, ^5^ Minniti et al^6^ found that HFSRT reduced the risk of radionecrosis as compared with SRS and associated with better local control. Lehrer et al^7^ made an international meta‐analysis of trials and found that HFSRT offered a relative reduction of radionecrosis while maintaining or improving 1‐year local control rate when compared to SRS. Therefore, HFSRT is effective to treat brain metastases, associates with better local control and reduces risk of radionecrosis as compared with SRS.^8^, ^9^, ^10^

Treatment planning of brain metastases is complicated because there are many critical and radiation‐sensitive structures including brainstem, eyes, and lenses in the brain. Therefore, a sharper dose falloff outside the targets is needed to protect organs at risk (OARs) and normal brain tissue better. There is a long history of conventional C‐arm based linear accelerator used to treat brain metastases. Beside conventional C‐arm based linear accelerator, there are some distinctive technologies, such as Gamma Knife (GK) and CyberKnife (CK). Due to non‐coplanar beams and the non isocentric nature of CK, the advantage of CK is quick dose falloff and better protection of normal brain tissue.^11^ CK contains a high‐resolution image‐guided tracking system to adjust the angle of beams during treatment to ensure the accuracy of the treatment. The shortcoming of GK and CK is that the treatment time is much longer than conventional C‐arm linear accelerator (linac) based plans such as intensity‐modulated radiation therapy (IMRT) and volumetric modulated arc therapy (VMAT).^12^ The dose falloff of IMRT and VMAT is not as quick as GK and CK. However, non‐coplanar technology has been used in C‐arm linac based plans to improve their dose falloff.

There are a lot of dosimetry comparison studies,^13^, ^14^ such as GK vs CK,^15^ GK vs coplanar VMAT,^16^ GK vs non‐coplanar VMAT,^17^, ^18^ coplanar IMRT vs coplanar VMAT,^19^ non‐coplanar IMRT vs coplanar VMAT,^20^ coplanar VMAT vs non‐coplanar VMAT.^21^ However, no direct comparison between CK and C‐arm linac based plans (including IMRT, coplanar VMAT and non‐coplanar VMAT) has been published. The aim of this study was to compare plan quality and delivery efficiency of CK and C‐arm linac based plans (IMRT, coplanar VMAT and non‐coplanar VMAT)for multiple brain metastases using HFSRT, to find the strengths and weaknesses of conventional C‐arm linac based plans and robotic radiosurgery.

## 2 METHODS

### 2.1 Patients

This study was approved by our Institutional Review Board, and written informed consent requirement was waived. All the image data were de‐identified by anonymization and analyzed retrospectively. There were 20 patients included in this study.

### 2.2 Treatment plans

All structures were delineated on Eclipse system (Varian Medical System Inc., USA, version 13.6). The image sets including all delineated structures were transferred via DICOM‐RT (Digital Imaging and Communications in Medicine–radiotherapy) to the CK Multiplan system (Accuray Inc., USA, version 4.6) for treatment planning. The planning target volume (PTV) and OARs, including brainstem, eyes, lenses, optic nerves, optic chiasm, pituitary,^22^ were delineated by an experienced radiation oncologist. Normal brain tissue was defined as healthy brain tissue minus PTV. The prescribed dose (D_p_) was 30 Gy in three fractions.^23^ The tolerance level of OARs for maximum dose was set according to TG101.^24^ The percentage of the PTV receiving at least the D_p_ was 95%.

#### 2.2.1 CK plan

The CK VSI radiosurgery system (Accuray Inc., USA) contains a compact 6‐MV linear accelerator and a computer‐controlled six‐axis robotic manipulator. The system combines a high‐resolution image‐guided tracking system to adjust the angle of beams during treatment to guarantee the accuracy of the treatment. In this study, CK plans were performed via skull tracking and RayTracing algorithm. More than one iris collimators were used for each plan. Collimators were chosen such that one collimator diameter was approximately equal to the central part of the largest lesion and the other was small enough to cover the tumor's smallest features. The therapeutic dose was prescribed to 70%–80% of the isodose line.

#### 2.2.2 C‐arm linear accelerator based plans

In this study, C‐arm linac based plans were designed based on TrueBeam linear accelerator at Eclipse system. These plans were created for delivery using the 6MV photon beam, with the Varian High Definition 120 multileaf collimator (MLC). All doses were calculated by means of an analytical anisotropic algorithm. The type of MLC motion was sliding window. The single isocenter was set at the center of mass of all brain metastases. The collimator angle was adjusted according to the location and size of the tumor in the Beam's Eye View.

IMRT plan was optimized with seven coplanar fields with a couch angle of 0°. VMAT plan consisted of two coplanar arcs of 356° optimized simultaneously and delivered with opposite rotation (clock and counter‐clock wise). The first arc started at a gantry angle of 182° and stopped at a gantry angle of 178°. The second arc started at a gantry angle of 178° and stopped at a gantry angle of 182°. The couch angle was set to 0° for both arcs.

Three types of non‐coplanar VMAT plans were designed in this study. The first non‐coplanar VMAT plan (NC1 plan) consisted of one full arc (couch angle: 0°) and one half arc (couch angle: 90°). The second non‐coplanar VMAT plan (NC3 plan) consisted of one full arc (couch angle: 0°) and three half arcs (couch angle: 45°, 90° and 315°). The third non‐coplanar VMAT plan (NC5 plan) consisted of one full arc (couch angle: 0°) and five half arcs (couch angle: 30°, 60°, 90°, 330° and 300°).

### 2.3 Evaluation tools

The gradient index (GI)^25^ described the steepness of the dose gradient from high (D_p_) to medium (50% of D_p_) dose levels. The conformity index (CI)^25^ was calculated to evaluate the degree of conformity of the dose distribution. The maximum dose was used to evaluate the dose to OARs. Normal brain tissue was evaluated by V_x Gy_. The indices were defined as follows:

1. GI = V_50% Dp_/V_p_, where V_50% Dp_ is 50% of the prescription isodose line volume, and V_p_ is the prescription volume.
2. CI = (V_tp_)^2^/(V_t_ × V_p_), where V_tp_ is the PTV volume within the prescribed isodose surface, V_t_ is the PTV volume and V_p_ is the prescription volume.
3. V_x Gy_ = V_x Gy_–V_t_, where V_x Gy_ is the volume receiving no less than x Gy, V_t_ is the PTV volume.

In addition, to investigate the efficiency of CK plan with respect to C‐arm linac based plans, delivery parameters were recorded in terms of monitor units (MUs) and beam on time (BT).

For each investigated parameter, significance of the differences observed among the techniques was computed with Wilcoxon signed rank test using Statistical Package for Social Science software (version 24.0, IBM SPSS Statistics) and the threshold was set to 0.05.

## 3 RESULTS

Twenty patients (median [range] age 67 [36‐81] years; 12 males and eight females) with 66 brain metastases were included in this study. The number of lesions ranged from 2 to 7 (two lesions: 9, three lesions: 4, four lesions: 1, five lesions: 5, seven lesions: 1). The volume of lesions ranged from 0.02 to 52.25 cm^3^, median 0.76 cm^3^. The total lesion volume for each patient ranged from 0.28 to 54.42 cm^3^, median 4.82 cm^3^.

**Figure 1:**
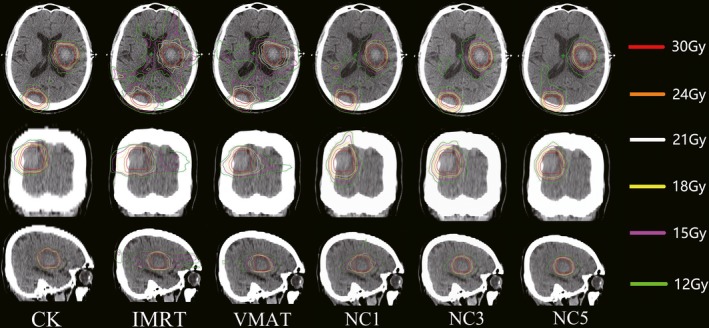
Dose distributions of CyberKnife (CK), intensity‐modulated radiation therapy (IMRT), volumetric modulated arc therapy (VMAT), non‐coplanar VMAT with one non‐coplanar arc (NC1), non‐coplanar VMAT with three non‐coplanar arc (NC3) and non‐coplanar VMAT with five non‐coplanar arc (NC5) plans in the axial plane (upper), coronal plane (center), and sagittal plane (lower) for a typical patient.

**Figure 2:**
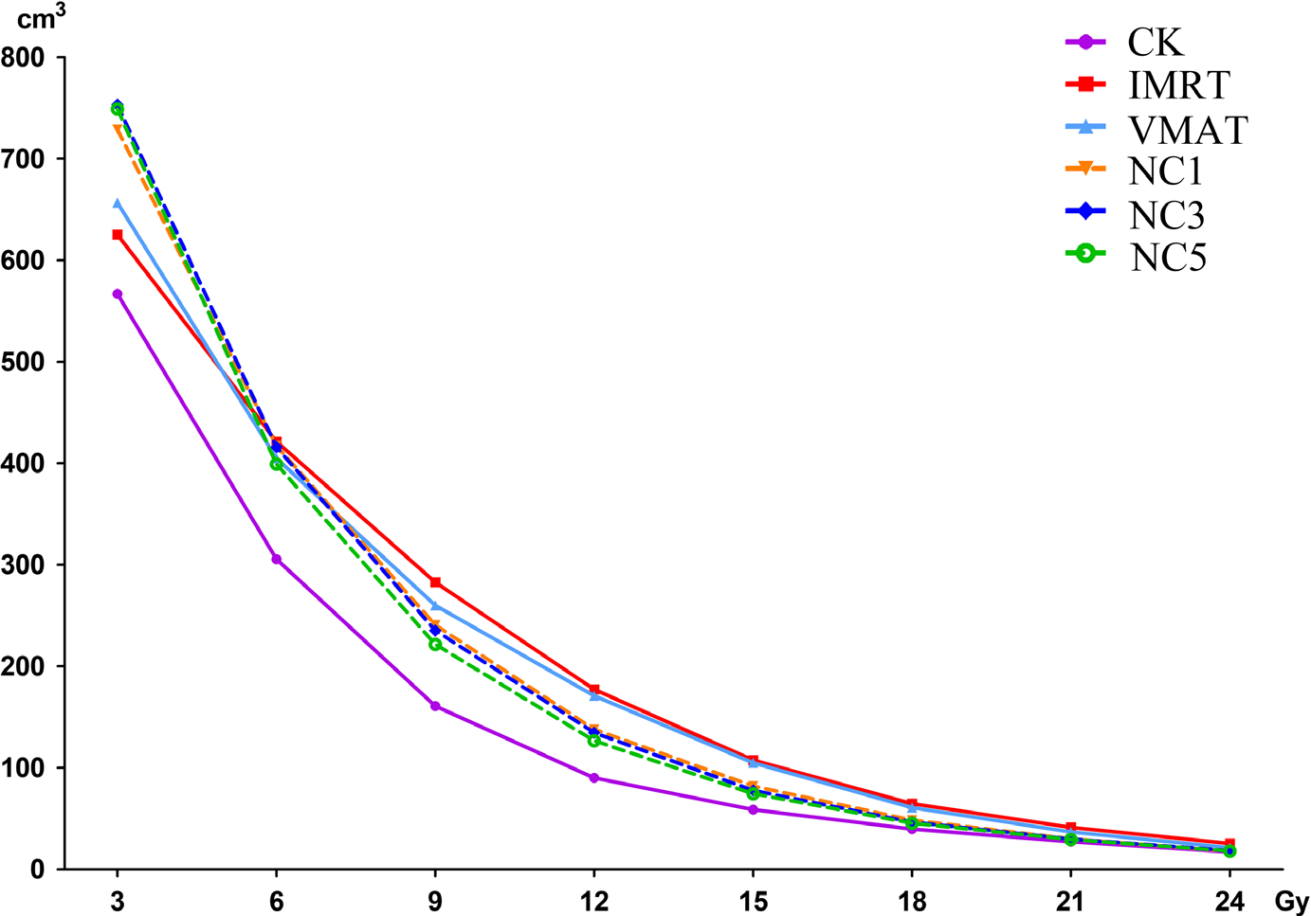
The average normal brain tissue volume receiving specific dose for CyberKnife (CK), intensity‐modulated radiation therapy (IMRT), volumetric modulated arc therapy (VMAT), non‐coplanar VMAT with one non‐coplanar arc (NC1), non‐coplanar VMAT with three non‐coplanar arc (NC3) and non‐coplanar VMAT with five non‐coplanar arc (NC5) plans.

Figure 1 presents the isodose distribution for a representative case (with four lesions) in three axial planes across the extension of the metastases for the six plans under investigation. From a qualitative perspective, the illustration showed the differences in terms of gradients and dose bridging mitigation among the various solutions. It could be observed that the CK plan provided a steeper dose gradient (V_12 Gy_ of this example for CK, IMRT, VMAT, NC1, NC3 and NC5 was 130.52, 303.87, 302.27, 206.97, 191.21 and 179.57 cm^3^ respectively). Figure 2 shows how the average normal brain tissue volume changed with dose. As Fig. 2 shows, the lines of IMRT, VMAT and the three non‐coplanar VMAT plans were higher than the line of CK, and the lines of the three non‐coplanar VMAT plans were very close. The lines of the three non‐coplanar VMAT plans were lower than the lines of IMRT and VMAT when the dose was larger than 6 Gy. However, they were higher than the lines of IMRT and VMAT when the dose was lower than 6 Gy.

Table 1 shows the dosimetric parameters and delivery parameters of the six plans. The GI was the lowest for CK plan, followed by NC3, NC5, NC1, VMAT and IMRT plans. The values of GI were comparable among the three non‐coplanar VMAT plans (*P* > 0.05). The CI of the VMAT plans (both coplanar and non‐coplanar) was larger than IMRT and CK plans. The MUs were the highest for CK plan, followed by IMRT, VMAT, NC1, NC3 and NC5 plans. There was no significant difference between IMRT and VMAT plans (*P* > 0.05). The BT was the longest for CK, followed by IMRT, VMAT, NC1, NC3 and NC5. Table 2 summarizes the maximum dose for OARs of the six plans. The maximum dose for most OARs of the CK plan was lower than the C‐arm linac based plans although some differences were not statistically significant. Table 3 shows the normal brain tissue volume receiving specific dose (24‐3 Gy) of NC1, NC3 and NC5 plans. The normal brain tissue volume for NC5 plan was lower than NC3 plan and NC1 plan (V_21 Gy_–V_6 Gy_) although some differences were not statistically significant. However, the average normal brain tissue volume receiving 3 Gy (V_3 Gy_) was the lowest for NC1 plan, followed by NC3 and NC5 plan.

**Table 1:** Evaluation parameters of CK, IMRT, VMAT, NC1, NC3 and NC5 plans ($\bar{x}$ ± SD).

Parameters	CK	IMRT	VMAT	NC1	NC3	NC5	*P*
GI	3.60 ± 0.68	6.21 ± 2.26	6.04 ± 1.93	5.16 ± 1.71	5.02 ± 1.59	5.03 ± 1.72	a, b, c, d, e
CI	0.86 ± 0.08	0.81 ± 0.07	0.90 ± 0.04	0.89 ± 0.07	0.89 ± 0.07	0.91 ± 0.07	a, b, c, d, e
MUs	29 031.96 ± 6562.18	3319.00 ± 1278.87	2968.00 ± 898.66	2527.90 ± 376.81	2518.25 ± 348.13	2384.95 ± 231.47	a, b, c, d, e
BT/min	39.05 ± 5.11	5.53 ± 2.13	4.95 ± 1.50	4.22 ± 0.63	4.20 ± 0.58	4.09 ± 0.29	a, b, c, d, e

Note

Statistical significance *P*: a = CK vs IMRT, b = CK vs VMAT, c = CK vs NC1, d = CK vs NC3, e = CK vs NC5.

Abbreviations: BT, treatment time; CI, conformity index; CK, CyberKnife; GI, gradient index; IMRT, intensity‐modulated radiation therapy; MUs, monitor units; NC1, non‐coplanar VMAT with one non‐coplanar arc; NC3, non‐coplanar VMAT with three non‐coplanar arc; NC5, non‐coplanar VMAT with five non‐coplanar arc; NS, not significant; VMAT, volumetric modulated arc therapy.

**Table 2:** The maximum dose for OARs of CK, IMRT, VMAT, NC1, NC3 and NC5 plans ($\bar{x}$ ± SD).

OAR	CK	IMRT	VMAT	NC1	NC3	NC5	*P*
Brainstem	4.22 ± 1.7	5.32 ± 2.37	5.09 ± 2.48	4.27 ± 1.28	3.66 ± 0.95	3.84 ± 0.92	a
Lens_R	0.30 ± 0.33	1.11 ± 0.83	1.07 ± 0.73	0.99 ± 0.33	0.97 ± 0.33	0.96 ± 0.30	a, b, c, d, e
Lens_L	0.19 ± 0.15	0.88 ± 0.83	1.12 ± 0.76	0.98 ± 0.43	0.96 ± 0.33	0.96 ± 0.30	a, b, c, d, e
Eye_R	1.72 ± 1.23	2.78 ± 1.68	2.29 ± 1.37	2.04 ± 0.64	2.03 ± 0.61	1.96 ± 0.60	a, b
Eye_L	1.55 ± 1.26	2.33 ± 1.72	2.25 ± 1.38	1.88 ± 0.94	1.90 ± 0.83	1.80 ± 0.73	a, b
Optic nerve_R	1.18 ± 1.04	1.77 ± 1.25	1.78 ± 1.16	1.74 ± 0.64	1.72 ± 0.65	1.78 ± 0.56	b, c, d, e
Optic nerve_L	1.09 ± 0.80	1.75 ± 1.65	1.60 ± 0.85	1.82 ± 0.73	1.73 ± 0.63	1.84 ± 0.52	b, c, d, e
Optic chiasm	1.96 ± 0.89	2.65 ± 1.71	2.32 ± 1.31	2.37 ± 0.65	2.25 ± 0.47	2.31 ± 0.47	NS
Pituitary	1.64 ± 0.73	1.94 ± 1.25	1.76 ± 0.95	1.71 ± 0.48	1.59 ± 0.39	1.82 ± 0.30	NS

Note

Statistical significance *P*: a = CK vs IMRT, b = CK vs VMAT, c = CK vs NC1, d = CK vs NC3, e = CK vs NC5.

Abbreviations: CK, CyberKnife; IMRT, intensity‐modulated radiation therapy; NC1, non‐coplanar VMAT with one non‐coplanar arc; NC3, non‐coplanar VMAT with three non‐coplanar arc; NC5, non‐coplanar VMAT with five non‐coplanar arc; NS, not significant; OARs, organs at risk; VMAT, volumetric modulated arc therapy.

**Table 3:** The normal brain tissue volume (receiving specific dose) of NC1, NC3 and NC5 plans ($\bar{x}$ ± SD).

Normal brain tissue volume	NC1	NC3	NC5	*P*
V_24 Gy_/cm^3^	17.69 ± 9.61	18.38 ± 10.26	18.15 ± 9.48	b
V_21 Gy_/cm^3^	29.9 ± 16.24	29.72 ± 16.48	29.33 ± 15.35	NS
V_18 Gy_/cm^3^	48.45 ± 28.28	46.43 ± 26.21	45.49 ± 24.65	b
V_15 Gy_/cm^3^	81.92 ± 52.03	77.74 ± 46.91	74.5 ± 43.21	b
V_12 Gy_/cm^3^	137.96 ± 91.07	134.66 ± 86.35	126.63 ± 78.96	b, c
V_9 Gy_/cm^3^	240.62 ± 160.65	235.2 ± 156.24	221.65 ± 144.33	b, c
V_6 Gy_/cm^3^	417.3 ± 252.41	415.78 ± 255.96	399.14 ± 252.78	c
V_3 Gy_/cm^3^	728.75 ± 291.03	753.14 ± 318.48	748.91 ± 330.21	NS

Note

Statistical significance *P*: a = NC1 vs NC3, b = NC1 vs NC5, c = NC3 vs NC5.

Abbreviations: NC1, non‐coplanar VMAT with one non‐coplanar arc; NC3, non‐coplanar VMAT with three non‐coplanar arc; NC5, non‐coplanar VMAT with five non‐coplanar arc; NS, not significant.

## 4 DISCUSSION

There are many treatment techniques for multiple brain metastases, each with its own characteristics.^26^, ^27^ In this study, we compared six plans including CK, IMRT, VMAT, NC1, NC3 and NC5. As the results indicate, the dose falloff of CK plan was evidently sharper than the C‐arm linac based plans. The dose falloff of the three non‐coplanar VMAT plans was similar and it was sharper than the coplanar plans (both IMRT and VMAT plans). They were observed in the GI and V_3 Gy_–V_24 Gy_ of normal brain tissue. Cao et al^11^ had compared dose metrics for six different plans. They found that the V_12 Gy_ of CK and GK plan was smaller than coplanar and non‐coplanar VMAT plans. Molinier et al^21^ had compared dose metrics for coplanar and non‐coplanar VMAT. They found that the V_10 Gy_ of non‐coplanar VMAT plan was smaller than coplanar VMAT for both single and multiple lesion cases. It illustrated that the plan with non‐coplanar beam, such as CK and non‐coplanar VMAT plans, had an advantage over decreasing normal brain tissue and protecting the cognition of patients with multiple brain metastases. As some patients needed to receive intracranial re‐irradiation due to intracranial recurrence, it seemed sensible to set the dose to the normal brain as low as possible.

In this study, the three non‐coplanar VMAT plans consisted of different numbers of non‐coplanar arcs. As shown in Fig. 2 and Table 3, the normal brain tissue volume of three non‐coplanar VMAT plans was similar. However, NC5 plan still had an advantage in protecting normal brain tissue compared to NC1 and NC3 plans at high‐moderate dose level (V_21 Gy_–V_6 Gy_). It illustrated that more non‐coplanar arcs could decrease normal brain tissue dose for multiple brain metastases. Hossain et al^28^ had compared the dose metric of three non‐coplanar VMAT plans with 3, 5 and 7 arcs, respectively. For multiple brain metastases, the normal brain tissue volume of non‐coplanar VMAT with 7 arcs was lower than non‐coplanar VMAT with 3 or 5 arc plans. Yoshio et al^29^ also found that non‐coplanar VMAT plan with 6 arcs could provide better protection for normal brain tissue than non‐coplanar VMAT plan with 4 arcs. However, at the low dose level (V_3 Gy_), the normal brain tissue volume of the NC5 plan was larger than NC1 and NC3 plans, and the normal brain tissue volume of all the three non‐coplanar VMAT plans was larger than coplanar plans (both IMRT and VMAT plans). It was because that beam of non‐coplanar arcs passed through a lot of normal brain tissue. The more non‐coplanar arcs the plan had, the more normal brain tissue the beam passed. Therefore, at the low dose level (V_3 Gy_), the normal brain tissue volume of the plan with more non‐coplanar arcs was larger than others.

The maximum doses of most OARs for CK plan were lower than the C‐arm linac based plans although some differences were not statistically significant (Table 2). It was because non‐coplanar beams were used in CK plan and the beams had more angles to choose to avoid passing OARs. Ning et al^30^ showed that VMAT plan with the use of non‐coplanar beams orientations significantly reduced peripheral doses when compared with coplanar VMAT. It illustrated that non‐coplanar radiation technology could reduce peripheral dose around PTV and protect OARs better.

In this study, all the C‐arm linac based plans were designed with a single isocenter. It was because that single isocenter VMAT plan was more efficient than multi‐isocenter VMAT plan with an equivalent conformity^31^. Uto et al^32^ had compared the dosimetric quality and delivery efficiency for two large brain metastases between dual‐isocentric dynamic conformal arc therapy (DCAT) with mono‐isocentric VMAT. They found that the treatment time of DCAT was longer than mono‐isocentric VMAT and concluded that it was because that there was no need to move the isocenters one after another in mono‐isocentric VMAT. The time for moving isocenter and the need for geometric verification at the other isocenter can be omitted in mono‐isocentric VMAT. Reduced treatment time could make the intrafractional error small, improve patients' throughput, and relieve patients' distress.

In this study, the C‐arm linac based plans were designed using Eclipse system, while CK plan was designed using Multiplan system. Different treatment planning system (TPS) may result in different plan quality and delivery efficiency. Ruggieri et al^33^ compared the plan quality and efficiency of two different TPS, Eclipse and MultipleBrainMets (Brainlab AG, Germany) for the treatment of multiple brain metastases. They found that the two TPS were comparable in GI, V_12 Gy_ and MUs. However, the CI of Eclipse plan was superior to MultipleBrainMets plan. Narayanasamy et al^34^ also found that the results of plans for the same case using different TPS were different.

## 5 CONCLUSION

CK plan was better than C‐arm linac based plans in protecting normal brain tissue and OARs for patients with multiple brain metastases. C‐arm linac based plans with non‐coplanar arc also provided better protection of normal brain tissue than coplanar plans. However, the BT of CK plan was longer than C‐arm linac based plans.

## CONFLICT OF INTEREST

The authors declare no conflict of interest.
